# Is telestroke more effective than conventional treatment for acute ischemic stroke? A systematic review and meta-analysis of patient outcomes and thrombolysis rates

**DOI:** 10.1177/17474930231206066

**Published:** 2023-10-27

**Authors:** Ahmed Mohamed, Salah Elsherif, Brittney Legere, Nida Fatima, Ashfaq Shuaib, Maher Saqqur

**Affiliations:** 1 Biology Department (Physiology), McMaster University, Hamilton, ON, Canada; 2 Department of Health Sciences, Queen’s University, Kingston, ON, Canada; 3 Department of Applied Sciences, University of Guelph, Guelph, ON, Canada; 4 Department of Neurosurgery, University of Texas Southwestern Medical Center, Dallas, TX, USA; 5 Department of Neurology, University of Alberta, Edmonton, AB, Canada; 6 Department of Neurology, University of Toronto, Mississauga, ON, Canada

**Keywords:** Telemedicine, telestroke, acute ischemic stroke, ischemia, thrombolysis

## Abstract

**Background:**

Telestroke systems operate through remote communication, providing distant stroke evaluation through expert healthcare providers. The aim of this study was to assess whether the implementation of a telestroke system influenced stroke treatment outcomes in acute ischemic stroke (AIS) patients compared with conventional in-person treatment.

**Aims:**

The study group evaluated multiple studies from electronic databases, comparing telemedicine (TM) and non-telemedicine (NTM) AIS patients between 1999 and 2022. We aimed to evaluate baseline characteristics, critical treatment times, and clinical outcomes.

**Summary of review:**

A total of 12,540 AIS patients were included in our study with 7936 (63.9%) thrombolyzed patients. Of the thrombolyzed patients, 4150 (51.7%) were treated with TM, while 3873 (48.3%) were not. The mean age of TM and NTM cohorts was 70.45 ± 4.68 and 70.42 ± 4.63, respectively (p > 0.05). Mean National Institute of Health Stroke Scale scores were comparable, with the TM group reporting a non-significantly higher mean (11.89 ± 3.29.6 vs. 11.13 ± 3.65, p > 0.05). No significant difference in outcomes was found for symptoms onset-to-intravenous tissue plasminogen activator (ivtPA) times (144.09 ± 18.87 vs. 147.18 ± 25.97, p = 0.632) and door-to-needle times (73.03 ± 20.04 vs. 65.91 ± 25.96, p = 0.321). Modified Rankin scale scores (0–2) were evaluated, and no significant difference was detected between cohorts (odds ratio (OR): 1.06, 95% confidence interval (CI): 0.89–1.29, p = 0.500). Outcomes did not indicate any significance between both cohorts for 90-day mortality (OR: 1.16, 95% CI: 0.94–1.43, p = 0.17) or symptomatic intracranial hemorrhage (OR: 0.99, 95% CI: 0.73–1.34, p = 0.93). Results between groups were also non-significant when analyzing the rate of thrombolysis with ivtPA (30.86%± 30.7 vs. 20.5%± 18.6, p = 0.372) and endovascular mechanical thrombectomy (11.8%± 11.7 vs. 18.7%± 18.9, p = 0.508).

**Conclusion:**

The use of telestroke in the treatment of AIS patients is safe with minimal non-significant differences in long-term outcomes and rates of thrombolysis compared with face-to-face treatment. Further studies comparing the different methods of TM are needed to assess the efficacy of TM in stroke treatment.

## Introduction

Telemedicine (TM) is the use of distant communication and information technologies to facilitate healthcare delivery for patients, helping physicians practice remotely.^1^ During the COVID-19 pandemic, the use of TM in healthcare significantly increased in various parts of North America.^2^ In the United States alone, telehealth visits increased from 13,000/week to 1.7 million/week post-COVID-19.^3^ More specifically, one study found that, after the pandemic, 83.1% of acute ischemic stroke (AIS) and transient ischemic attack patients had one or more TM visits within 90 days of emergency department discharge, compared with 3.8% before the pandemic.^4^ With such rapid increases, TM in stroke (telestroke) may be an effective tool to support the efficient delivery of healthcare services from remote locations.

The benefits of integrating telestroke into AIS patient care are immense. The implementation of this novel technology application has increased accessibility to healthcare services for patients in remote and rural areas.^5,6^ Telestroke can also reduce the cost of healthcare by eliminating the necessity of travel and provides positive outcomes for patients.^7^ Despite these positive outcomes, the challenge remains of ensuring telestroke patients receive the same effectiveness of therapy as traditionally treated patients. Time delays in AIS patients’ treatment may result in a higher risk of brain tissue injury, cell death, and cerebral infarction.^8^ Research suggests a 10% decrease in the chance of obtaining favorable outcomes with each 15-minute delay after event onset.^8^

In this systematic review, we aim to identify if the application of TM as a treatment method for AIS patients will improve clinical outcomes, functional independence, and critical times compared with traditional in-person treatment of AIS patients.

## Methods

### Data search strategy

The Preferred Reporting Items for Systematic Review and Meta-analysis (PRISMA) was followed to inform the literature search strategy^9^. Two reviewers (SE and AM) performed a thorough systematic review and screening of various studies from different electronic databases including EMBASE, PubMed, Google Scholar, and Cochrane Library. Studies included were published between January 1, 1990, and December 31, 2022. The relevant searched MeSH terms included the following keywords: “Telestroke,” “thrombolysis,” “door-to-needle,” “stroke,” “tissue plasminogen activator,” “thrombectomy,” and “face-to-face stroke treatment.”

### Data extraction

Three authors participated in statistical analysis (AM, SE, and BL) and data collection from online sources. All conflicts between the authors were resolved by discussion and meetings. Data collected from the studies included (1) mean age and sex distribution of participants in each study, (2) number of participants treated with TM versus non-telemedicine (NTM) controls, (3) number of patients thrombolyzed and number of patients treated with mechanical thrombectomy (MT), (iv) mean door-to-needle times (DTN) and mean symptoms onset-to-intravenous tissue plasminogen activator (ivtPA), and (v) types of neuroimaging used in AIS patients.

In addition, various clinical outcomes were also reported including discharge National Institute of Health Stroke Scale (NIHSS) scores, the number of patients who experienced symptomatic intracranial hemorrhage (sICH) after stroke occurrence, stroke-related mortality at 90 days, and modified Rankin scale (mRS) scores at 90 days. A good outcome was defined as one having values between 0 and 2 on the mRS scale, while values between 3 and 6 were considered poor functional outcomes. A value of 6 was defined as mortality.

Primary analysis of the outcomes included the mRS values for the AIS patients, which were used as a major determinant of clinical outcomes. Secondary analysis included mortality, critical times including DTN, mean symptoms onset to ivtPA, and other events (Table 2).

### Inclusion and exclusion criteria

Randomized controlled trials (RCTs) as well as retrospective and prospective studies comparing the clinical outcomes among patients treated either through TM or conventional face-to-face stroke treatment were included. Case reports, case series, and case–control studies were all excluded from the review analysis.

### Statistical analysis

All statistical analysis comparing data from included studies was conducted with Rev Manager version 5.3. Dichotomous data were analyzed using odds ratio (OR), while pooled weight mean difference was used to analyze the continuous data. The results were reported as either OR or mean difference. Secondary analyses for critical time parameters between TM and NTM groups were conducted using multiple paired t-tests. I^2^ statistics were used to evaluate the heterogeneity among the studies. The fixed effect model was used for I^2^ < 50%, while, for I^2^ > 50%, a random-effect model was employed. All tests were two-tailed, and p value ⩽ 0.05 was considered statistically significant.

### Risk of bias across studies

Two randomized control trials were included in this study; however, none of them were designed as double-blind trials. The high heterogeneity was analyzed using the funnel plot. It showed asymmetrical distribution, which may be attributed to a small sample size as the removal of a small-sized cohort significantly decreased the heterogeneity.

## Results

### Study selection

**Figure 1.**
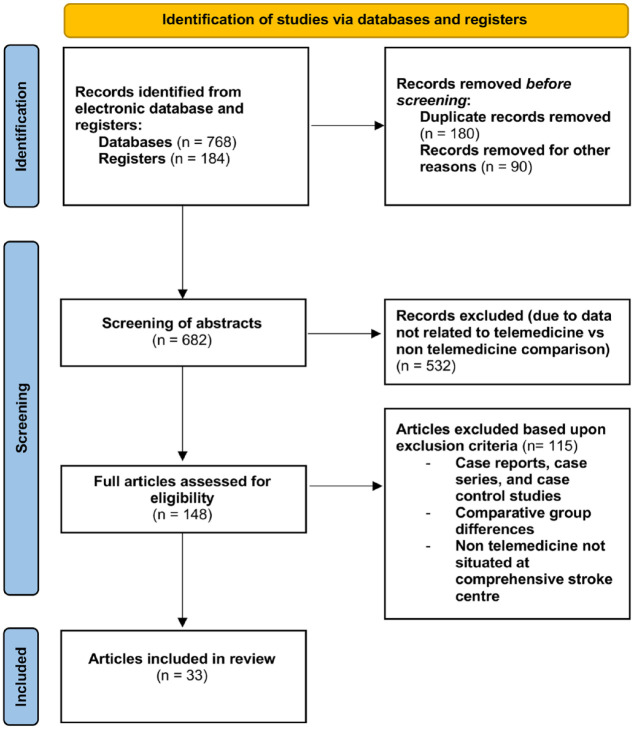
Preferred Reporting Items for Systematic Review and Meta-analysis (PRISMA) flowchart.

Articles were reviewed according to PRISMA guidelines (Figure 1); a total of 952 articles were retrieved from electronic databases. Five hundred and thirty-two articles were excluded due to the inclusion of data unrelated to TM or NTM and a lack of comparison between both cohorts. A further 116 articles were excluded as inclusion criteria were not met. These studies included case reports, case series, comparative group differences, and studies where NTM patients were not situated at the comprehensive stroke center and had to be transported (due to the potential of skewing critical times data).

Of the 148 remaining articles, 33 were investigated further and included 20 retrospective studies,^10–29^ 10 prospective studies,^30–40^ and 2 RCTs.^41,42^ These studies were conducted in the United States (n = 15),^10,11,16,17,20–22,24,25,27–29,33,35,38,40^ Germany (n = 4),^12,30,31,34,41^ France (n = 4),^15,32,42^ the United Kingdom (n = 3),^13,18,26^ Hong Kong (n = 1),^19^ Spain (n = 2),^23,37^ Italy (n = 1),^36^ Austria (n = 1),^14^ and Finland (n = 1).^39^

### Characteristics and critical times of the study

A total of 12,540 AIS patients were included in our analysis, of which 7936 patients were thrombolyzed. Some studies only reported the number of thrombolyzed patients, providing no data for any remaining non-thrombolyzed AIS patients. Of the thrombolyzed patients, 3873/7936 (48.3%) were in the NTM control group, with 4150/7936 (51.7%) in the TM group. The mean age for analyzed patients was 70.45 ± 4.68 and 70.42 ± 4.63 years for the TM and NTM groups, respectively. Minimal differences were also observed in baseline NIHSS scores, with reported mean scores of 11.89 ± 3.29.6 and 11.13 ± 3.65 for the TM and NTM groups, respectively (p > 0.05). The baseline characteristics of the included studies are outlined in Table 1.

**Table 1.** Baseline characteristics, critical times, rates of thrombolysis, and thrombectomy of all patients in the study.

	Location	Type of study and number of AIS patients	Mean age in years (range)		Mean NIHSS at baseline		Sex composition		Number of patients treated with telemedicine (TM)	Number of patients treated without telemedicine (NTM)	Number of patients thrombolyzed		Mean door-to-needle time in minutes		Mean symptoms onset to IVtPA time in minutes		Type of neuroimaging used in AIS patients	Number of patients treated with mechanical Thrombectomy	
			TM	NTM	TM	NTM	TM	NTM			TM	NTM	TM	NTM	TM	NTM		TM	NTM
Alkasab et al.^10^—Pre	United States	Retrospective NA	65.5	64.6	NA	NA	274 Females (51.9%)	81 Females (48.50%)	528 (75.97%)	167 (24.03%)	695	90.1	62.3	NA	NA	NA	NA	NA	
Alkasab et al.^11^—Post	United States	Retrospective NA	66.2	66.2	NA	NA	360 Females (45.3%)	90 Females (51.4%)	795 (81.95%)	175 (18.05%)	970	64.6	46.1	NA	NA	NA	NA	NA	
Audebert et al.^12^	Germany	Retrospective 3889	69.7 ± 10.7	69.8 ± 11.4	12.4 ± 4.9	11.9 ± 5.3	51 Females (44.35%)	41 (37.27%)	2603 (66.9%)	1286 (33.1%)	115 (4.4%)	110 (8.6%)	68 ± 23	61 ± 23	134 ± 30	135 ± 38	CT scan	NA	NA
Chowdhury et al.^13^	United Kingdom	Retrospective NA	62.51 ± 11.52	64.46 ± 12.00	13.45 ± 7.37	14.81 ± 6.98	17 Females (37.7%)	23 Females (44.2%)	45 (46.39%)	52 (53.61%)	97	61	33	125 (55–105) **δ**	100 (78–120) **δ**	NA	NA	NA	
Eder et al.^30^	Germany	Prospective 496	NA	NA	NA	NA	112 Females (45.0%)	110 Females (44.9%)	250 (50.40%)	246 (49.60%)	100 (40%)	125 (50.8%)	35 (27–46) **δ**	39 (28–49) **δ**	NA	NA	CT scan	NA	NA
Eder et al.^31^	Germany	Prospective 1,124	78.8 (70.3–85.1) **δ**	78.5 (68.8–85.2) **δ**	6 (3–11) **δ**	5 (2–10)**δ**	229 Females (50.4%)	358 Females (53.5%)	455 (40.48%)	669 (59.52%)	173 (38%)	183 (27.4%)	25 (20–35) **δ**	29 (23–40) **δ**	NA	NA	CT scan	NA	NA
Johansson et al.^14^	Austria	Retrospective NA	67 ± 15	71 ± 16	9.9 ± 5.2	10.4 ± 5.9	31 Females (66%)	152 Females (50%)	47 (13.39%)	304 (86.61%)	351	NA	NA	113 ± 40	122 ± 47	MRI +CT scan	NA	NA	
Kaminsky et al.^32^	France	Prospective 207	74.5 ± 15.2	72.8 ± 13.5	17.0 (13.5–20.0) **δ**	16.0 (10.0–20.0) **δ**	44 Females (58.7%)	76 Females (57.6%)	75 (36.23%)	132 (63.77%)	61 (81.3%)	71 (53.8%)	NA	NA	171.4 ± 47.3	166.4 ± 48.7	MRI + CT scan	20 (26.7%)	65 (49.2%)
Pedragosa et al.^33^	United States	Prospective NA	71.6	71	19 **δ**	18.5 **δ**	8 Females (30.1%)	41 Females (55.1%)	25 (25.3%)	74 (74.7%)	99	46	100	143 ± 65	131 ± 74	CT scan +carotid ultrasound	NA	NA	
Raulot et al.^15^	France	Retrospective NA	75 ± 15	74 ± 12	12 (9–19) **δ**	14 (8–18) **δ**	16 Females (59.2%)	32 Females (45.7%)	27 (27.8%)	70 (72.2%)	97	86 (63–94) **δ**	55 (45–64) **δ**	180 (158–208)**δ**	170 (135–199)**δ**	MRI +CT scan	NA	NA	
Shwab et al.^34^	Germany	Prospective NA	69.4	69.6	12 (2–25) **δ**	11 (2–34) **δ**	69 Females (40.6%)	48 Females (36.4%)	170 (56.29%)	132 (43.71%)	302	NA	NA	140.6	143.6	CT scan +MRT	NA	NA	
Sobhani et al.^16^	United States	Retrospective 132	73.1 ± 13.8	79.2 ± 11.6	3.5 ± 4	4 ± 6	29 Females (52.78%)	40 Females (51.9%)	55 (41.67%)	77 (58.33%)	3 (3.9%)	7 (12.7%)	56.8	41.5	121.7	108.5	CT scan	0 (0%)	0 (0%)
Switzer et al.^35^	United States	Prospective NA	63	NA	14.4	NA	29 Females (60%)	NA	49 (65.3%)	26 (34.7%)	75	NA	NA	127.5 ± 36.33	145.8 ± 46.99	CT scan	NA	NA	
Amorim et al.^17^	United States	Retrospective 2,588	73.2 ± 13.8	73.9 ± 11.5	12 **δ**	8 **δ**	58 Males (51.3%)	14 Males (51.9%)	1669 (64.49%)	919 (35.51%)	113 (6.77%)	27 (2.94%)	74 ± 29.1	74.2 ± 32.1	124.4 ± 34	129.8 ± 34	CT scan	NA	NA
Dutta et al.^18^	England	Retrospective NA	76	78	12.5 **δ**	12 **δ**	108 Males (49.1%)	190 Males (51.9%)	220 (37.5%)	366 (62.5%)	586	76.6 (72.2–80.9)	53.7 (50.9–56.5)	161.6 (154.9–168.3)	139.5 (134.5–144.4)	CT scan	NA	NA	
Ebinger et al.^41^	Germany	Randomized Controlled Trial 1,655	76.7 ± 12.4	74.9 ± 13.1	10.5	9.2	92 Males (46%)	108 Males (49.1%)	614 (37.1%)	1041 (62.9%)	200 (32.6%)	220 (21.1%)	NA	36	102.7 (93.9–111.5)	118.5 (111.8–125.2)	CT scan	NA	NA
Fong et al.^19^	Hong-Kong	Retrospective NA	65.3 ± 10.3	67 ± 11.8	12.5 (7.8–19)	12 (8–19)	32 Males (64%)	56 Males (54.9%)	50 (32.9%)	102 (67.1%)	152	97 (85–119)	71 (60–89)	148 (134–170)	133 (109–154)	CT scan	NA	NA	
Frey et al.^20^	United States	Retrospective NA	67.02 (36–89) **δ**	61.71 (29–92) **δ**	NA	NA	34 Males (62.2%)	35 Males (47.9%)	53 (42.1%)	73 (57.9%)	126	NA	NA	NA	NA	CT scan, CT angiogram, CT brain scan	NA	NA	
Ionita et al.^21^	United States	Retrospective NA	72.3 ± 15.1	71.4 ± 16.9	12.2 ± 6.4	12.6 ± 4.9	14 Males (52%)	57 Males (45%)	27 (17.4%)	128 (82.6%)	155	NA	NA	130.7 ± 42.1	143.9 ± 29.5	CT scan	NA	NA	
Mansoor et al.^22^	United States	Retrospective NA	62.5 ± 13.2	60.1 ± 14.6	12 **δ**	11 **δ**	14 Males (54%)	94 Males (54%)	26 (12.9%)	175 (87.1%)	201	85	80	151.8 ± 91.5	152.9 ± 126.3	MRI + CT scan + MRA	NA	NA	
Martinez-Sanchez et al.^23^	Spain	Retrospective 484	72.2 ± 12.5	70.5 ± 12.6	6.5 (8) **δ**	6.5 (9) **δ**	112 Males (49.8%)	132 Males (51%)	225 (46.5%)	259 (53.51%)	18 (8%)	12 (4.63%)	66 **δ**	143.5 **δ**	155 **δ**	205 **δ**	CT scan	0 (0%)	1 (8.3%)
Martin-Schild et al.^24^	United States	Retrospective 428	65 ± 16	65 ± 15	10.7 ± 5.8	13.1 ± 6.7	37 Males (32%)	139 Males (44.6%)	116 (27.1%)	312 (72.9%)	84 (72.4%)	NA	85 **δ**	64 **δ**	150 **δ**	135 **δ**	MRI + CT scan	NA	NA
Mazighi et al.^42^	France	Randomized Controlled Trial 47	80 (23–92) **δ**	71 (22–89) **δ**	13 (4–22) **δ**	7 (4–17) **δ**	8 Males (32%)	7 Males (31.8%)	25 (53.2%)	22 (46.8%)	21 (84%)	4 (18.2%)	NA	NA	150 ± 22.5	156.5 ± 20	CT scan	NA	NA
Nardetto et al.^36^	Italy	Prospective NA	68.5 ± 10.7	67.8 ± 14.4	10.4 ± 5	11.6 ± 6.1	NA	NA	25 (19.1%)	106 (80.9%)	131	73 **δ**	95 **δ**	151.4 ± 44.1	165.88 ± 44.2	CT scan	NA	NA	
Pedragosa et al.^37^	Spain	Prospective 399	75 ± 8	68 ± 13	18 (11–19)	19 (17–20)	NA	NA	198 (65.8%)	201 (34.2%)	19 (9.60%)	9 (4.48%)	NA	NA	162 ± 84	210 ± 43	CT scan	NA	NA
Pervez et al.^25^	United States	Retrospective NA	71.5 ± 14.7	73.6 ± 12.4	13 (7–18) **δ**	12 (8–19) **δ**	47 Males (25.96%)	56 Males (48.7%)	181 (61.15%)	115 (38.85%)	296	NA	NA	140.88 ± 35.5	131.7 ± 44.6	CT scan	NA	NA	
Qureshi et al.^38^	United States	Prospective NA	71.6 ± 13.6	69.7 ± 15.9	NA	NA	NA	NA	129 (21.4%)	473 (78.6%)	602	NA	NA	NA	NA	CT scan	NA	NA	
Rudd et al.^26^	United Kingdom	Retrospective NA	75 (25–92) **δ**	76 (47–97) **δ**	14 (4–24) **δ**	13.5 (3–24) **δ**	NA	NA	94 (52.8%)	84 (47.2%)	178	73 (51–95) **δ**	65 (46–84) **δ**	NA	NA	CT scan	NA	NA	
Sairanen et al.^39^	Finland	Prospective 1091	72 **δ**	70 **δ**	10 (8) **δ**	10 (9) **δ**	30 Males (49.1%)	535 Males (54.3%)	106 (5.8%)	985 (94.2%)	61 (57.5%)	NA	NA	NA	NA	NA	CT scan	NA	NA
Sorensen et al.^27^	United States	Retrospective NA	69.1 ± 14.7	73.6 ± 15.1	10 (15–18) **δ**	6 (3–13) **δ**	135 Males (49.6%)	129 Males (48.3%)	272 (50.5%)	267 (49.5%)	539	100.6 ± 46.7	82.8 ± 50.5	174.1 ± 83.1	176 ± 126.8	NTM (noncontrast head CT, CT perfusion, CT angiography) TM (noncontrast head CT only) Follow up for both (MRI and CT)	39 (14.3%)	37 (13.9%)	
Uchino et al.^28^	United States	Retrospective NA	66.4 ± 16	70.7 ± 13.3	NA	10	NA	NA	133 (60.73%)	86 (39.27%)	219	90 (70–110)	78 (60–99)	142 (116.5–174.25)	149 (120–167)	CT scan	24 (18%)	19 (22%)	
Yaghi et al.^29^	United States	Retrospective NA	67 ± 14	67 ± 16	12 ± 7	11 ± 6	73 Males (52%)	16 Males (35%)	141 (75.4%)	46 (24.6%)	187	91 ± 28	72 ± 32	156 ± 44	154 ± 54	MRI, Head CT scan (patients w pacemakers)	NA	NA	
Zaidi et al.^30^	United States	Prospective NA	71.9 ± 14.4	71.9 ± 14.1	12 (4–33) **δ**	10.5 (2–38) **δ**	44 Males (53.1%)	26 Males (43.6%)	83 (58.5%)	59 (41.5%)	142	89.9 ± 36.3	67.8 ± 26.1	145.5 ± 42.8	156.7 ± 31.6	CT scan	NA	NA	

AIS: acute ischemic stroke; NIHSS: National Institute of Health Stroke Scale; TM: telemedicine; NTM: non-telemedicine; MRA: magnetic resonance angiography; CT: computed tomography; δ: Median Value.

Variances in critical time measurements (symptoms onset to ivtPA and DTN) between both groups for thrombolyzed patients are also presented in Table 1. Although the mean symptoms onset to ivtPA time for the NTM group was higher than the TM group, no statistical significance was presented (147.18 ± 25.97 min vs. 144.09 ± 18.87 min, p = 0.6327). Similarly, DTN times for the TM group were not significantly greater than the NTM group (73.03 ± 20.04 min vs. 65.91 ± 25.96 min, p = 0.321). Although most studies included in our systematic review reported comparable values for DTN times between both TM and NTM groups, 2 studies reported NTM DTN values almost 2-fold the times presented by the TM group: 100 min versus 46 min^33^ and 143.5 min versus 66 min.^23^ Similar substantial values were reported for symptom onset to ivtPA times for the Martinez-Sanchezf^23^ study with mean values of 205 and 155 min for the NTM and TM groups, respectively.

### Clinical outcomes of studies

**Figure 2.**
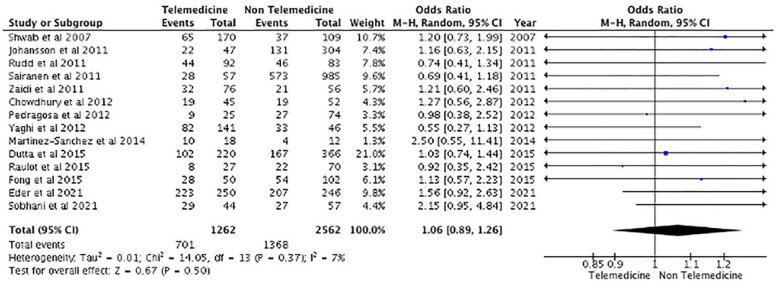
Good functional outcome (modified Rankin scale (mRS) = 0–2) at 90 days.

Clinical outcome characteristics are described in Table 2. The mRS is reported for all studies 90 days following the onset of symptoms. Excluding certain studies,^10,11,12,17,20–22,25,27,28,31,35–38,41,42^ the pooled analysis of clinical outcomes at day 90 indicates that patients treated using the TM care model had an equivalent likelihood of scoring a good clinical outcome, indicated by an mRS score of 0–2, compared with those in the NTM group. Between the two cohorts, the mRS score results were not statistically significant (OR: 1.06, 95% confidence interval (CI): 0.89–1.29, p *=* 0.50) (Figure 2). Similarly, the results show that the number of patients scoring mRS (3–6) was not significant when comparing the TM and NTM models of care.

**Table 2.** Clinical outcomes 90 days post-operation.

Name of study	Rate of successful recanalization		Discharge NIHSS scores Mean ±SD (range)		Symptomatic intracranial hemorrhage		90-day stroke-related mortality		mRS scores at 90 Days (0–2)		mRS scores at 90 Days (3–6)		Type of neuroimaging used in AIS patients
	TM	NTM	TM	NTM	TM	NTM	TM	NTM	TM	NTM	TM	NTM	
Alkasab et al.^10^—Pre	NA	NA	NA	NA	NA	NA	NA	NA	NA	NA	NA	NA	NA
Alkasab et al.^11^—Post	NA	NA	NA	NA	NA	NA	NA	NA	NA	NA	NA	NA	NA
Audebert et al.^12^	NA	NA	NA	NA	9/115 (7.8%)	3/110 (2.7%)	19/170 (11.2%)	15/132 (11.3%)	NA	NA	NA	NA	CT scan
Chowdhury et al.^13^	NA	NA	NA	NA	2/45 (4.4%)	4/52 (7.7%)	8/45 (17.8%)	5/52 (9.6%)	19/45 (42%)	19/52 (36.5%)	26/45 (58%)	33/52 (63.5%)	NA
Eder et al.^30^	NA	NA	NA	NA	1/250 (0.4%)	2/246 (0.8%)	1/250 (0.4%)	1/246 (0.41%)	223/250 (89.6%)	207/246 (84.5%)	27/250 (10.8%)	39/246 (15.9%)	CT scan
Eder et al.^31^	NA	NA	NA	NA	NA	NA	15/455 (3.3%)	18/669 (2.7%)	NA	NA	NA	NA	CT scan
Johansson et al.^14^	NA	NA	NA	NA	NA	NA	9/47 (19%)	40/304 (13%)	22/47** (47%)	131/304** (43%)	NA	NA	MRI + CT scan
Kaminsky et al.^32^	NA	NA	NA	NA	19/75 (25.4%)	57/132 (43.3%)	28/75 (37.2%)	33/132 (25.2%)	24/75 (32.1%)	46/132 (35.1%)	51/75 (67.9%)	86/132 (64.9%)	MRI + CT scan
Pedragosa et al.^33^	17/25 (69%)	55/74 (74%)	NA	NA	2/25 (8%)	4/74 (5.4%)	NA	NA	9/25 (35.3%)	27/74 (36.8%)	16/25 (64%)	47/74 (63.5%)	CT scan + carotid ultrasound
Raulot et al.^15^	NA	NA	10/27 (2–20) (Median)	8/70 (3–16) (Median)	1/27 (4%)	5/70 (7%)	8/27 (29.6%)	22/70 (31%)	8/27 (30%)**	22/70 (31.4%)**	NA	NA	MRI + CT scan
Shwab et al.^34^	NA	NA	NA	NA	NA	NA	NA	NA	65/170 (38.2%)**	37 (33.7%)**	NA	NA	CT scan + MRT
Sobhani et al.^16^	NA	NA	NA	NA	NA	NA	NA	NA	29/44 (65.9%)	27/57 (47.3%)	15/44 (34.1%)	30/57 (52.7%)	CT scan
Switzer et al.^35^	NA	NA	NA	NA	2/49 (4.1%)	0/26 (0%)	NA	NA	NA	NA	NA	NA	CT scan
Amorim et al.^17^	NA	NA	NA	NA	1/113 (0.9%)	1/27 (3.7%)	NA	NA	NA	NA	NA	NA	CT scan
Dutta et al.^18^	NA	NA	NA	NA	8/220 (3.6%)	17/366 (4.6%)	33/220 (15%)	64/366 (17.5%)	102/220 (46.0%)	167/366 (46.1%)	118/220 (54%)	199/366 (53.9%)	CT scan
Ebinger et al.^41^	NA	NA	NA	NA	7 (3.5%)	14 (6.4%)	33/200 (16.5%)	27/220 (12.2%)	NA	NA	NA	NA	CT scan
Fong et al.^19^	NA	NA	NA	NA	2/50 (4%)	5/102 (4.9%)	4/50 (8%)	12/102 (11.8%)	28/50 (58.3%)	54/102 (54.0%)	22/50 (41.7%)	48/102 (46%)	CT scan
Frey et al.^20^	NA	NA	NA	NA	1/53 (1.9%)	0/73 (0%)	NA	NA	NA	NA	NA	NA	CT scan, CT angiogram, CT brain scan
Ionitta et al.^21^	NA	NA	NA	NA	9 (33%)	26 (20%)	NA	NA	NA	NA	NA	NA	CT scan
Mansoor et al.^22^	NA	NA	NA	NA	3/26 (11.5%)	11/175 (6.3%)	NA	NA	NA	NA	NA	NA	MRI + CT scan + MRA
Martinez-Sanchez et al.^23^	NA	NA	NA	NA	0/18 (0%)	0/12 (0%)	NA	NA	10 (55.6%)**	4 (33.3%) **	NA	NA	CT scan
Martin—Schild et al.^24^	NA	NA	NA	NA	5/84 (5.9%)	14/312 (4.5%)	NA	NA	12 (37.5%)**	21 (62.5%) ***	MRI + CT scan		
Mazighi et al.^42^	NA	NA	NA	NA	1/25 (4%)	0/22 (0%)	NA	NA	NA	NA	NA	NA	CT scan
Nardetto et al.^36^	NA	NA	NA	NA	2/25 (8%)	3/106 (2.83%)	NA	NA	NA	NA	NA	NA	CT scan
Pedragosa et al.^37^	NA	NA	5 (1–15)	4 (1–17)	0/19 (0%)	0/9 (0%)	NA	NA	NA	NA	NA	NA	CT scan
Pervez et al.^25^	NA	NA	NA	NA	7/181 (3.9%)	6/115 (5.2%)	NA	NA	NA	NA	NA	NA	CT scan
Qureshi et al.^38^	NA	NA	NA	NA	NA	NA	NA	NA	NA	NA	NA	NA	CT scan
Rudd et al.^26^	NA	NA	NA	NA	0/80 (0%)	2/67 (2.9%)	NA	NA	44/92 (46.8%)	46/83 (55.4%)	48/92 (53.2%)	37/83 (44.6%)	CT scan
Sairanen et al.^39^	NA	NA	NA	NA	NA	NA	7/61 (11.5%)	100/985 (10.15%)	28/57 (49.1%)	573/985 (58.1%)	29/57 (50.9%)	412/985 (41.9%)	CT scan
Sorensen et al.^27^	NA	NA	NA	NA	14/272 (5.2%)	7/267 (2.6%)	NA	NA	NA	NA	NA	NA	NTM (noncontrast head CT, CT perfusion, CT angiography) TM (noncontrast head CT only) Follow up for both (MRI and CT)
Uchino et al.^28^	NA	NA	NA	NA	9/133 (6.8%)	5/86 (5.8%)	NA	NA	NA	NA	NA	NA	CT scan
Yaghi et al.^29^	NA	NA	NA	NA	4/141 (2.8%)	1/46 (2.2%)	NA	NA	82/141 (58.2%)	33/46 (70%)	59/141 (42%)	13/46 (30%)	MRI, Head CT scan (patients w pacemakers)
Zaidi et al.^40^	NA	NA	NA	NA	1/83 (1.2%)	3/59 (5.1%)	26/83 (31.3%)	18/59 (30.5%)	32/76 (42.1%)	21/56 (37.5%)	44/76 (57.9%)	35/56 (37.5%)	CT scan

** = mRS 0-1; *** = good outcome defined as discharge home or to inpatient rehabilitation; AIS: acute ischemic stroke; NIHSS: National Institute of Health Stroke Scale; TM: telemedicine; NTM: non-telemedicine; MRA: magnetic resonance angiography; CT: computed tomography.

**Figure 3.**
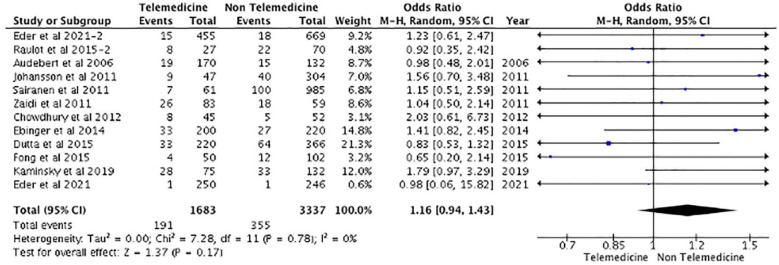
Mortality at 90 days.

**Figure 4.**
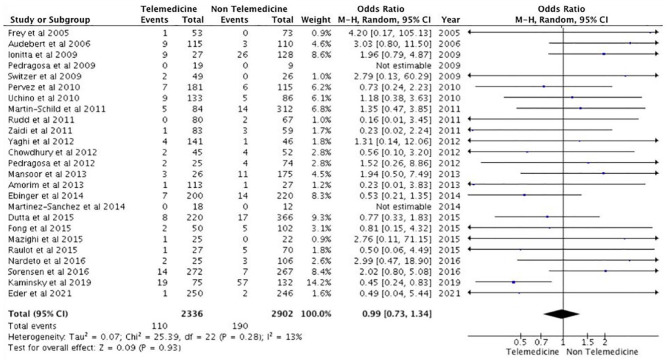
Symptomatic intracranial hemorrhage (sICH) at 90 days.

Only one study^33^ reported the rate of successful recanalization. Pedragosa et al.^33^ reported 69% and 74% for TM and NTM cohorts, respectively. Similarly, only two studies^15,37^ reported discharge NIHSS scores, indicating no statistical significance between both cohorts. There was also no significant difference reported in terms of 90-day stroke-related mortality among patients treated using the TM model of care or NTM model of care (OR: 1.16, 95% CI: 0.94–1.43, p = 0.17) (Figure 3). Furthermore, although the number of individuals experiencing sICH post-treatment is larger in the TM group in some studies,^12,20–22,24,27–29,33,35,36,42^, this was not sufficient to show a significant difference (OR: 0.99, 95% CI: 0.73–1.34, p = 0.93) (Figure 4). Finally, the rates of thrombolysis and thrombectomy did not significantly differ between the two groups (p = 0.372 and p = 0.508, respectively). The TM cohort reported a 30.86%± 30.7 thrombolysis rate and a 11.8%± 11.7 MT rate, while the NTM cohort reported a 20.5%± 18.6 thrombolysis rate and a 18.7%± 18.9 MT rate.

## Discussion

TM is recommended worldwide as an effective and safe form of decision-making regarding thrombolysis.^43^ Our systematic review and meta-analysis revealed that TM is as effective as NTM in providing acute stroke care and management with non-significant higher rates of IV thrombolysis; this is primarily in telestroke sites where a specialist is not available and acute stroke care can be offered by TM with a drip-and-ship (DS) model. Patients with large vessel occlusion (LVO) often bypass primary stroke centers, getting transported directly to comprehensive centers (mothership model (MS)). This may explain the non-significant higher rate of EVT in the NTM group. Patients with a low probability of LVO are most likely to get treated in a primary stroke center by TM. The distinction between treating AIS patients with the TM model of care in comparison with the NTM model of care is that patients arriving in hospitals without the specialist on site may utilize various forms of virtual care delivery to obtain guidance from the consulting physician.

The overall results of the study show no differences between TM and NTM groups through the course of stroke treatment. Insignificant differences in DTN times between TM and NTM patients may suggest that telestroke technology can efficiently facilitate stroke treatment response and administration, mainly in the primary stroke center where the neurologist or stroke specialist is not available in person.

The study’s results offer a continuation of Baratloo’s review, comparing the effects of the implementation of TM on AIS outcomes.^44^ Our data offer a deeper insight into stroke patient outcomes, thrombolysis rates, and MT with a higher number of patients analyzed. Through remote consultations with stroke specialists, patients treated via TM may develop trust in the efficacy of telestroke care, which can be particularly important for populations residing in areas with limited access to healthcare professionals. Similarly, results comparing the time of symptom onset to ivtPA treatment among both groups were insignificant, further corroborating the benefit of telemedical techniques. Since the time between the onset of symptoms to the administration of ivtPA is a critical window for successful stroke treatment and TM patients were treated within a similar timeframe, the study can confirm that the presence of TM has no difference in the ability to facilitate initiation of treatment.^45^ The critical time result differences in Pedragosa et al.^33^ and Martinez-Sanchez et al.^23^ between the TM and NTM cohorts may be explained through these studies’ implementation of the DS model for the telestroke program, which has been proven by previous literature to instill longer symptom onset to puncture times.^46^

Our study further compared the clinical outcomes and functional independence between both cohorts. Only one study^33^ reported the rate of successful recanalization, making it challenging to compare the effect of TM on this variable. The discharge NIHSS score was also only reported in three studies.^15,26,37^ Considering the percentages of patients in both groups experienced sICH after their treatment, rates were comparatively close and typically fluctuated between a range of 0% and 10%. Two studies^21,32^ reported a rate of sICH higher than 10% for both groups, while one study^21^ reported a rate higher than 10% for the TM group only.

Of the 12 studies^12–15,18,19,30–32,39–41^ that reported 90-day stroke mortality, the values were in similar ranges with 8 out of 13 studies demonstrating a non-significant higher mortality in TM care.^13,14,31,32,39–41^ Similarly, no statistical significance was found in mRS scores 90 days post-stroke between TM and NTM care. However, of the 17 studies^13–16,18,19,23,24,26,29,30,32–34,39,40^ that reported mRS scores 90 days post-stroke, patients using NTM care tended to have a non-significantly higher likelihood of scoring poor clinical outcomes, indicated by an mRS score of 3–6. Out of the 17 studies, 5 reported mRS scores of 0–1;^14,15,23,24,34^ as such, poor functional independence could not be calculated for patients in these studies. Furthermore, only one study^24^ reported mRS scores for TM patients. Therefore, clinical outcomes between TM and NTM patients could not be compared.

Finally, the rate of patients thrombolyzed in the studies varied over a wide range. In the TM cohort, some studies, such as Audebert et al.^12^ and Sobhani et al.,^16^ reported lower percentages of thrombolyzed patients (4.4% and 3.9%, respectively). Meanwhile, other studies such as Kaminsky et al.^32^ and Mazighi et al.^42^ reported rates of 81.3% and 84%, respectively. The NTM cohort, like the TM cohort, also experienced a large range of thrombolyzed patients. Studies reported rates starting at 2.94%,^17^4.48%,^37^ and 4.63%^23^ up until 27.4%^31^ and 53.8%.^32^

Our analysis demonstrated no significant difference in the rate of thrombolysis or thrombectomy between TM patients in comparison with NTM. Rates of thrombolysis were non-significantly higher in the TM group compared with the NTM group. Perhaps this may be a selection bias whereby only patients who were candidates for thrombolysis may have been captured in the consultation between the TM physician and the consultant at the tertiary care center. In addition, a trend was present toward increasing MT rates for the NTM group in comparison to the TM group. Although non-significant, this trend may be due to a variety of factors, including differences in patient selection criteria, greater access to medical facilities, and more frequent use of advanced imaging. The MS sends patients directly to a comprehensive stroke center for treatment allowing for a faster arrival time to the comprehensive stroke center and more drastic treatment intervention.^46^

Our study also showed the different types of imaging techniques utilized to treat ischemic stroke patients, whether by using TM or NTM methods. Most of the articles included in the included studies utilized computed tomography (CT) scans, while others combined CT scans with magnetic resonance imaging (MRI). It is possible that the use of advanced imaging may provide patients with better information on brain tissue status. Despite this, no differences were observed in studies utilizing CT imaging alone versus CTA and MRI.^24,25,37^

There are a few limitations in our study. Most of the studies analyzed in this systematic review did not specify the form of TM used in the TM model of care groups. The form of TM used (e.g., video vs. phone call) may result in longer process times. This confounding variable was not adjusted in our analysis due to the lack of data provided. Another limitation is that patients in TM and NTM groups may have used different transport models that were not accounted for in this systematic review. MS and DS models may impact patient outcomes differently.^47^ As highlighted above when examining Martinez-Sanchez et al.,^23^ DS models may result in longer critical times which could affect the results of this review and produce poorer outcomes in patients, regardless of TM usage. Since data acquisition may have been different among care providers, our study cannot rule out the presence of information bias as well. Furthermore, only three studies analyzed discharge NIHSS scores and only one study reported the rate of successful recanalization. Finally, there are multiple ways to mechanistically evaluate the etiology of the stroke, where CT angiography (CTA) and magnetic resonance angiography (MRA) may be useful. Only two studies reported using CTA and one reported the use of MRA. Therefore, no further analysis was completed on this topic due to the unavailability of data, and we were unable to predict whether it could have shown a better outcome.

Further studies are needed comparing TM and NTM groups with regard to the different patient transport models to create an optimal treatment plan, validate the potential advantages or disadvantages of TM usage, and minimize poor outcomes for AIS patients. Another point of future analysis may be to investigate the use of advanced imaging such as CTA and MRA to further evaluate patient outcomes and revascularization success.

## Conclusion

The use of telestroke in the treatment of AIS patients is safe considering the non-significant differences in long-term outcomes, thrombolysis, and MT rates compared with face-to-face treatment. Our results provided considerable insight into the efficacy of telestroke systems and presented an understanding for future directions in optimizing patient care. Further studies evaluating the types of patient transport models and types of TM will be needed to ensure proper assessment of the implementation of TM.
